# Incorporating basic and applied approaches to evaluate the effects of invasive Asian Carp on native fishes: A necessary first step for integrated pest management

**DOI:** 10.1371/journal.pone.0184081

**Published:** 2017-09-05

**Authors:** Quinton E. Phelps, Sara J. Tripp, Kyle R. Bales, Daniel James, Robert A. Hrabik, David P. Herzog

**Affiliations:** 1 West Virginia University, Division of Forestry and Natural Resources, 322 Percival Hall Morgantown, WV; 2 Missouri Department of Conservation, 3815 East Jackson Boulevard, Jackson, Missouri; 3 Iowa Department of Natural Resources, 206 Rose St., Bellevue, Iowa; 4 United States Fish and Wildlife Service, Great Plains Fish and Wildlife Conservation Office Pierre, South Dakota; University of Windsor, CANADA

## Abstract

Numerous studies throughout North America allege deleterious associations among invasive Asian Carp and native fishes; however, no empirical evidence on a system-wide scale exists. We used Mississippi River Basin fish community data collected by the Long Term Resource Monitoring program and the Missouri Department of Conservation to evaluate possible interaction between Asian Carp and native fishes. Results from two decades of long-term monitoring throughout much of the Mississippi River suggest that Silver Carp relative abundance has increased while relative abundance (Bigmouth Buffalo [F _3, 8240_ = 6.44, P<0.01] and Gizzard Shad [F _3, 8240_ = 31.04, P<0.01]) and condition (Bigmouth Buffalo [slope = -0.11; t = -1.71; P = 0.1014] and Gizzard Shad [slope = -0.39; t = -3.02; P = 0.0073]) of native planktivores have declined. Floodplain lake qualitative evaluations yielded similar results; floodplain lake fish communities were likely altered (i.e., reductions in native species) by Silver Carp. Furthermore, laboratory experiments corroborated field evidence; Silver Carp negatively influence native planktivores through competition for prey (all comparisons, *P* > 0.05). To this end, this study provides evidence that Silver Carp are likely adversely influencing native fishes; however, mere presence of Silver Carp in the system does not induce deleterious effects on native fishes. To the best of our knowledge, this evaluation is the first to describe the effects of Asian Carp throughout the Mississippi River Basin and could be used to reduce the effects of Asian Carp on native biota through an integrated pest management program as suggested by congressional policy. Despite the simplicity of the data analyzed and approach used, this study provides a framework for beginning to identify the interactions of invasive fish pests on native fishes (i.e., necessary first step of integrated pest management). However, knowledge gaps remain. We suggest future efforts should conduct more in depth analyses (i.e., multivariate statistical approaches) that investigate the influence on all native species.

## Introduction

Intentional and accidental introduction of species outside of their native range occur without full comprehension of their effects on native fauna [1–3]. Introduced species have the potential to pose undesirable, unintended consequences on ecosystems and associated native biota through transformation of basic ecosystem structure and function. To combat these deleterious effects, integrated pest management programs have been employed in numerous countries to control or eradicate nuisance species [4–6]. Integrated pest management utilizes a multi-tiered approach that encompasses prevention, early detection measures, monitoring, and containment or control tools to exploit pest species of interest [6], [7]. At its onset, integrated pest management combined chemical and biological methods to counteract the negative impacts of pest insects in agriculture systems [4], [8], [9]. More recently, integrated pest management has evolved into a more robust technique applied to a broader range of species and ecosystems [3], [10], [11].

As it relates to fishes, integrated pest management has been used to combat the increased successful introductions of non-native fishes [12–14]. Barriers, such as, acoustical deterrence, bubble barriers, and CO2 chambers have all been researched with the hope to reduce the spread of non-native fishes. For example, Vetter et al. [15] found that Silver Carp in cement ponds avoided complex sounds but did not react to pure tones. Furthermore, in order to implement successful integrated pest management plans, baseline species-specific information (e.g., biology and ecology) is needed [12]. However, only in some cases have the relative effects of introduced fishes on native fish populations and the associated aquatic communities been formally documented [1], [16]. In many systems, the ecological niches of native and invasive fishes are similar, creating potentially limited resources and subsequent competitive interactions initiating declines in native fish populations [13], [17]. In cases where prey overlap exists between native and non-native fishes, reductions in prey availability negatively influence growth and survival of native fishes [18–20]. Understanding the effects of introduced species on native fish communities is critical in developing the baseline information needed to develop measures of an integrated pest management program.

Of the many fish species invading novel waters, Asian Carps are among the most introduced and subsequently invasive fishes in North America [7]. The threat of Asian Carps (i.e., Bighead Carp *Hypophthalmichthys nobilis*, Black carp *Mylopharyngodon piceus*, Grass Carp *Ctenopharyngodon idella*, and Silver Carp *Hypophthalmichthys molitrix*) on inland North American waters have dramatically increased. Asian Carps were introduced into the United States for human consumption and supposed biological control [13], [21–23]. Asian carps have expanded their range and have established populations in many U.S. freshwater lentic and lotic environments [13], [21], [22]. Like many other successful invaders, Asian carp persist in many locations because they possess “r-selected” characteristics (e.g., rapid growth, fast dispersal capabilities, high reproductive potential, absence of natural predators, and broad environmental tolerance [13], [24]).

Because of these attributes Asian Carp often attain high densities, can have injurious effects (e.g., harmful) on aquatic environments [17], [21], [22], and are costly to control [25]. Because of these potential injurious effects, congressional policy has recently been put in place to regulate Asian carp populations. Specifically, the Water Resources Reform and Development Act was signed into law on June 10, 2014 by the President. As a part of this act, the United States Fish and Wildlife Service in association with the National Parks Service and United States Geological Survey are to take actions on the expanding Asian Carp in the Mississippi River Basin and its tributaries. The report suggests incorporating an integrated pest management plan for Asian Carp in locations throughout the Mississippi River Basin. The integrated pest management plan must use the most current comprehensive scientific information available as it relates to the influence or interactions that these highly invasive species poses to the environment. However, a comprehensive understanding of the effects that Asian carp have on aquatic ecosystems in the United States is limited and direct evidence of the effects on a broad spatiotemporal scale does not exist.

One of the most detrimental and ubiquitous invasive Asiatic fish species is Silver Carp. Silver Carp are a large planktivorous species [26], [27] native to many of the major Pacific drainages of eastern Russia south to northern Vietnam [13]. Silver Carp were brought to the United States to facilitate biological control and thus enhance water quality in aquaculture facilities in the 1970s [21]. After escaping such facilities, Silver Carp spread throughout the Mississippi River Basin and now inhabit many of its tributaries [21], [22]. Since that time, Silver Carp abundance has increased substantially and may be posing negative effects on native aquatic organisms. Planktivorous invasive species like the Silver Carp may compete with native species on a small scale [19], but this interaction has not been directly evaluated on a system wide basis [28].

These perceived harmful interactions are likely driven by the exceedingly high abundance or biomass, migrate long distances, jumping ability (danger to boaters) and their effective filter-feeding ability [19], [29]. Because of this extreme filtering efficiency, Silver Carp have the capability to diminish planktonic resources in areas occupied [30–36]. Additional studies suggest that native fishes may be negatively affected through declines in body condition due to increasing numbers of these invasive filter feeders [19]; (D. Chapman, United States Geological Services, Personal Communication). Although direct competition between native fishes and Silver Carp is important, negative effects have not been documented and has been reported as a research need [19]. Direct competition only occurs when species share a common resource that is limited and fitness is reduced [37].

The objective of this study was to determine population level interactions of Silver Carp with native fishes. We used data collected by the Long Term Resource Monitoring program (LTRM) and the Missouri Department of Conservation in the Mississippi River basin from 1993–2012. We also sampled four Mississippi River floodplain lakes having varying abundances of Silver Carp to evaluate the response in fish community composition after Silver Carp invaded during and after the flood of 2011. Controlled experiments were conducted to determine if competition was structuring the relationship between Silver Carp and native fishes. This study aims to provide evidence that would help facilitate the development of integrated pest management program on Asian carp in North America. Furthermore, our study provides a framework for identifying the interactions of invasive fish pests on native fishes (i.e., a critical component of integrated pest management), regardless of location.

## Methods

### Ethics statement

The Illinois and Mississippi River are public waterways, requiring no specific permission to access them. Fish were collected by state agencies located in each of the pools listed below. Fish were collected throughout Pool 4 (Lake City, Minnesota, RKM 1210–1283, Coordinates 44.4493^o^N, 92.2667^o^W), Pool 8 (LaCrosse, Wisconsin, RKM 1092–1131, Coordinates 43.8014^o^N, 91.2396^o^W), Pool 13 (Bellevue, Iowa, RKM 841–896, Coordinates 42.2589^o^N, 90.4258^o^W), Pool 26 (Alton, Illinois, RKM 325–389, Coordinates 38.8906^o^N, 90.1843^o^W), La Grange Pool, Illinois River (Havana, Illinois, RKM 128–252, Coordinates 40.3000^o^N, 90.0610^o^W), and the Open River (Cape Girardeau, Missouri, RKM 47–129, Coordinates 37.3059^o^N, 89.5181^o^W). All collected fish were held in stock tanks and worked up as quickly as possible to reduce stress and mortality per Long Term Resource Monitoring element and Missouri Department of Conservation veterinary protocols. Once measured and weighed fish were returned alive to the river from which they were collected. All collections were approved by Long-Term Resource Monitoring Program and corresponding state agency authorities.

### Long-term field data collection

This portion of the study was conducted in 6 reaches of the Mississippi River Basin; Pool 4, Pool 8, Pool 13, Pool 26, La Grange Pool, Illinois River, and the Open River. From the northern uppermost reach (Lake City, Minnesota) to the southern lowermost reach (Cape Girardeau, Missouri) longitudinal variation in environmental attributes exists. Vegetation density and physical complexity are greater in the upper reaches species relative to the lower reaches [38], [39]. Fish species richness also differs between locations, but differences are minor [39]. These locations were sampled using standardized LTRM electrofishing from 1993–2012 between early June and the end of October for fish community trend monitoring where Silver Carp, Bigmouth Buffalo *Ictiobus cyprinellus*, and Gizzard Shad *Dorosoma cepedianum [40],* [41]. Total lengths (mm) were recorded for each captured fish. Mean catch-per-unit-effort (CPUE, recorded as total number of individuals per species captured per hour of electrofishing) by year and location for each of the above species were calculated. These data were used to analyze trends over time, and to monitor the pre- and post-Silver Carp invasion.

The three uppermost reaches, Pool 4, Pool 8, and Pool 13, have not been fully invaded by Silver Carp and serve as a control reach while the three lower reaches, Pool 26, La Grange, and Open River, have well established Silver Carp populations and serve as an impact reach. Although environmental variability exists longitudinally, electrofishing is performed during the same time period among all reaches and is accounted for given the statistical analyses performed. We used a ‘Beyond BACI’ (Before-After-Control-Impact) study design [42] to evaluate the effect of Silver Carp where they have become established. This approach employs replicated temporal and spatial sampling of multiple control sites compared to multiple impacted sites to test for the effects of an environmental disturbance, such as invasive species introductions [42]. Sampling at each control and treatment site occurred for ten years before and for ten years after Silver Carp were established between 1993 and 2012. As such, this approach accounts for pre-existing (i.e., before) differences in control and impact locations. Conditions present prior to the impact in both locations are used as the benchmark to evaluate whether the environmental perturbation has influenced either location. For example, in cases where no statistical differences exist in the control locations while statistical differences are noted in the impact locations, this indicates that the environmental perturbation is having an effect.

An asymmetrical analysis of variance (AANOVA) is used for this BACI design to determine the significance of possible effects of Silver Carp establishment [42–45]. In this analysis contrasts of the impacted versus control locations and their interactions with time are obtained from the variation within each control and impacted location and their interaction with time [42–44]. A sequence of tests were conducted following [45] to determine if Silver Carp establishment resulted in a change in CPUE in the treatment areas of the two response variables (Gizzard Shad and Bigmouth Buffalo CPUE) that differed from the pattern in control locations. Three results are possible with this analysis: 1) no impact, 2) a pulse impact, or 3) a press impact [44]. No impact indicates that Silver Carp did not cause a change in pattern (e.g., Silver Carp establishment did not impact Gizzard Shad or Bigmouth Buffalo CPUE) at the treatment sites compared with the control locations. A pulse or press impact would indicate that Silver Carp establishment caused a change in CPUE at a treatment site (e.g., Gizzard Shad or Bigmouth Buffalo mean CPUE experienced a short- or long-term increase or decrease) compared with the control locations [42], [44]. Because weights were only collected from a subsample of Bigmouth Buffalo, Gizzard Shad, and Silver Carp during the LTRM evaluation (see above), additional weight data were recorded in the Open River reach between early June and the end of October from 1993–2012 to evaluate condition.

Condition indices, relative weight or Fulton’s condition factor, were computed using weights from Bigmouth Buffalo (n = 2,124), Gizzard Shad (n = 4,287), and Silver Carp (n = 2,764) in the Open River reach to evaluate the potential influence of Silver Carp on Bigmouth Buffalo and Gizzard Shad. Slope tests within linear regression analyses were conducted to determine whether the relationship between the condition indices for each species and the sampling year were different from zero.

### Floodplain lake evaluation

The floodplain evaluation took place in the New Madrid Floodway (Coordinates 36.7798^o^N, 89.3856^o^W) adjacent to the Mississippi River. The floodway has an area of approximately 536 square km and begins just south of Cairo, IL and extends southward to New Madrid, MO. The Floodway is approximately 53 kilometers long, with a maximum width of 16 kilometers, all of which was inundated during the flood of 2011 after the levee was breached by the United States Army Corps of Engineers to relieve flooding pressure. The New Madrid floodway had not been inundated since 1937. Since 1937, none of the floodplain lakes sampled had ever been inundated by the Mississippi River. After the 2011 flood, four floodplain lakes similar in size (>2.5 ha) and water quality characteristics (temperature and dissolved oxygen) were sampled using standardized LTRM electrofishing protocols [41]; (Table 1). Extensive electrofishing (i.e., the entire shoreline was sampled) occurred after the river became disconnected to the floodplain lakes in early June and again in early November. During both sampling events on each lake, Silver Carp CPUE was generated and presence absence of all other species was recorded. Each of the four floodplain lakes were then categorized using a similar approach to Fremling et al. [46] and Schramm et al. [47] based on Silver Carp CPUE (not detected [0/hr], low [10/hr], moderate [100/hr], and high [>100/hr]). Changes in fish communities were compared with presence/not detected data using only commonly collected (i.e., more than 10 of that species were collected with electrofishing) taxa during our first and last sampling events.

**Table 1:** Floodplain lake data. Description of the floodplain lakes sampled during June and November of 2011 within the New Madrid Floodway each with varying levels of Silver Carp invasion based on standardized electrofishing.

Floodplain Lake	Level of Invasion	Shoreline Length (m)	Area (ha)	Average Depth (m)		Effort (min/sampling event)
A	Not Detected	582	1.9	3.0		22
B	Low	549	1.7	2.2		20
C	Moderate	627	1.7	2.8		25
D	High	890	2.4	3.2		30

### Controlled laboratory experiments

We evaluated interactions between Silver Carp, Bigmouth Buffalo, and Gizzard Shad in a controlled environment at the Big Rivers and Wetlands Field Station in Jackson, Missouri to evaluate interspecific and intraspecific interactions among these species. Age-0 Silver Carp, Bigmouth Buffalo, and Gizzard Shad were collected using seines from the Mississippi River and its floodplain and were allowed to acclimate under laboratory conditions for one week prior to experimental trials. Laboratory experiments were performed using 128 identical 37.9 liter covered glass aquaria in rows of eight where temperature (20 ±1°C) and lighting (mimicked the natural light-dark cycle) were kept constant. Each aquarium had sand substrate and water physical attributes such as volume, temperature, and dissolved oxygen were also kept constant. After the acclimation period, all fish were individually marked with fin clip, weighed, and equal densities (N = 4 fish/tank) of fishes were randomly placed in each aquarium.

We performed 128 trials to evaluate both interspecific interactions (Silver Carp *Gizzard Shad (N = 25), and Silver Carp *Bigmouth Buffalo (N = 26) and intraspecific interactions (Silver Carp*Silver Carp (N = 25), Gizzard Shad*Gizzard Shad (N = 26), and Bigmouth Buffalo*Bigmouth Buffalo (N = 26)) for this portion of the study. For interspecific interactions equal densities of Silver Carp (N = 2) were put into an aquarium with the same density of Gizzard Shad (N = 2) or Bigmouth Buffalo (N = 2). Experiments were also conducted to evaluate intraspecific interactions using the same densities as noted above (N = 4). All fish (34mm to 123mm) within each experiment were within 0.2 g and similar total lengths of their counterparts’ weights and lengths. Fishes were fed daily a maintenance ration (1% body weight / day) of freshly collected plankton. Plankton were collected from the same locations where experimental fish were captured. Total amount or wet weight of plankton provided to each aquarium was based on the entire initial weight of all fishes within each experimental aquarium. Fish mortalities were recorded daily. At the end of the 14-d trials, survival and growth for each species were evaluated. We compared survival and growth (g) differences between interspecific and intraspecific groups using paired t-tests.

## Results

### Long-term field data

**Fig 1:**
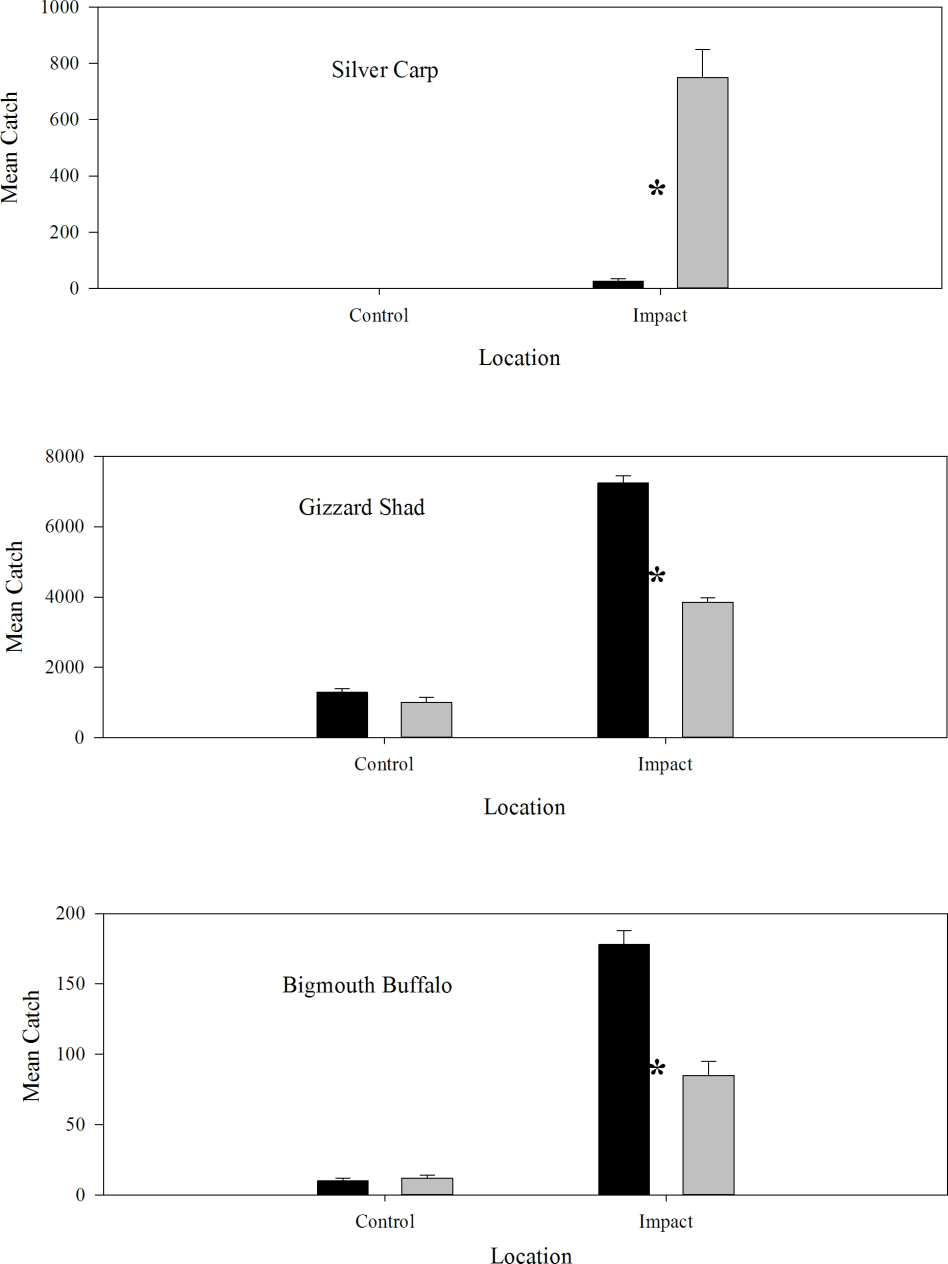
Control and Impact areas before and after 2003. Pool 4 (Lake City, Minnesota, RKM 1210–1283), Pool 8 (LaCrosse, Wisconsin, RKM 1092–1131), Pool 13 (Bellevue, Iowa, RKM 841–896)) versus impact locations (Pool 26 (Alton, Illinois, RKM 325–389), La Grange Pool, Illinois River (Havana, Illinois, RKM 128–252), and the Open River (Cape Girardeau, Missouri, RKM 47–129) in terms of electrofishing mean catch per unit effort (Mean Catch; number/hour) before (black bars) and after 2003 (gray bars). * Denotes treatments were statistically different, P <0.05. Standard error bars are present.

**Fig 2:**
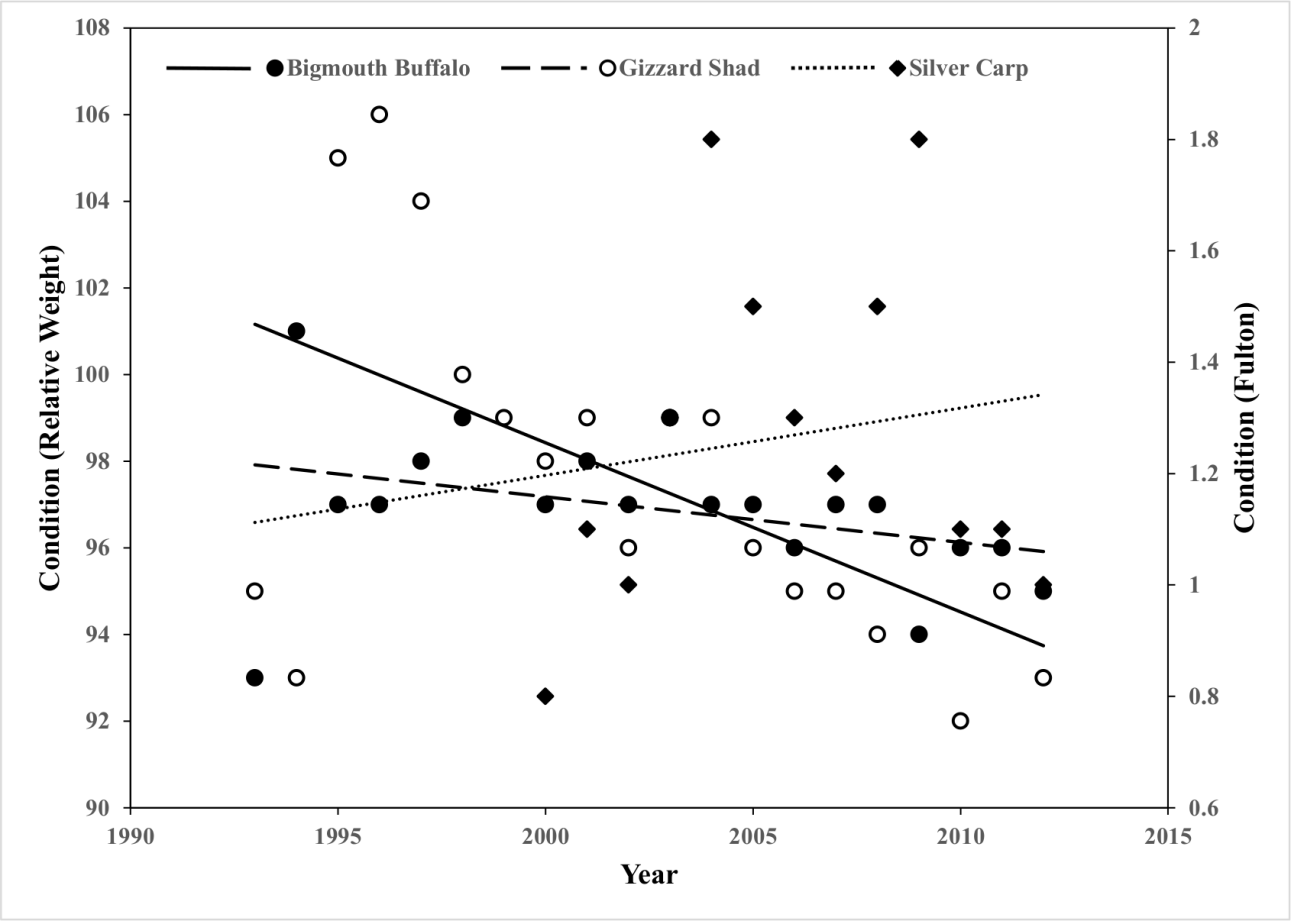
Condition indices and Fulton’s condition factor. Condition indices (i.e., relative weight [Bigmouth Buffalo and Gizzard Shad] and Fulton’s condition factor [Silver Carp]) computed in the lower most reach (Open River, Cape Girardeau, Missouri, RKM 47–129) of the Mississippi River from 1993–2012.

The AANOVA indicated a press impact or a long term decrease in CPUE for both Gizzard Shad and Bigmouth Buffalo (Table 2; Fig 1). In the control locations Gizzard Shad mean CPUE did not change from the before to after time periods; CPUE was 1,368/hr before and 1,008/hr after (Table 2; Fig 1). However, mean CPUE significantly decreased and remained depressed after Silver Carp invasion at impacted sites; CPUE decreased from 7,186/hr to 3,810/hr (Table 2; Fig 1). For Bigmouth Buffalo, the same pattern was apparent. At control locations, Bigmouth Buffalo CPUE was 11 before and 12 after. However, mean CPUE decreased from 178 to 85 at sites impacted by Silver Carp invasion (Table 2; Fig 1). These results suggest that the CPUE of Gizzard Shad and Bigmouth Buffalo have declined over time while Silver Carp CPUE has increased. We also found that Silver Carp condition has remained fairly consistent (slope = 0.01; t = -0.48; P = 0.6207) in the Open River reach over time while Bigmouth Buffalo (slope = -0.11; t = -1.71; P = 0.1014) and Gizzard Shad condition (slope = -0.39; t = -3.02; P = 0.0073) declined between 1993–2012, suggesting potential resource limitation in the Mississippi River for the native fishes (Fig 2).

**Table 2:** Summary of asymmetrical analyses of variance. Summary of asymmetrical analyses of variance comparing catch per unit effort of Gizzard Shad and Bigmouth Buffalo from the Mississippi River Basin from before and after Silver Carp invasion. Final results are determined from a sequence of tests described in [44].

Species	Short-term variation in control reach after invasion	Invasion affects short-term temporal variation	Differences in controls from before to after invasion	Invasion affects long-term temporal variation	Final Result
Gizzard Shad	F _18, 8240_ = 0.16	F _27, 8240_ = 1.06	F _2, 8240_ = 0.25	F _3, 8240_ = 6.44	press impact
	*p* = 1.00	*p* = 0.39	*p* = 0.78	*p* < 0.01	
Bigmouth Buffalo	F _18, 8240_ = 0.05	F _27, 8240_ = 0.91	F _2, 8240_ = 0.33	F _3, 8240_ = 31.04	press impact
	*p* = 1.00	*p* = 0.60	*p* = 0.72	*p* < 0.01	

### Floodplain lake evaluation

During our first sampling event, we collected 27 dominant fish taxa among the four floodplain lakes. An overwhelming majority of the fishes captured were young-of-the-year based on total length [48]. Silver Carp were collected in three of the four floodplain lakes. After the approximate five-month time frame between the first sampling event and the last sampling event, changes were observed in some lakes. No changes in fish community occurred over the five-month period in the Floodplain Lake A, where Silver Carp were absent. In contrast Floodplain Lake D with high Silver Carp abundance displayed a drastically altered floodplain lake fish community. The two other floodplain lakes had low and moderate abundances of Silver Carp. These lakes displayed minimal changes to the floodplain lake fish community. However, the Floodplain Lake C with moderate abundance of Silver Carp had some minor changes with a reduction in the abundance of two native species over the five-month duration. Overall, we have shown with this simplistic approach that as Silver Carp abundance increases, the native fish community can be drastically altered in floodplain lakes over time (Table 3).

**Table 3:** A qualitative comparison of change in fish communities within floodplain lakes. A qualitative comparison of change in fish communities within floodplain lakes of varying Silver Carp abundances using presence/not detected data. Only commonly collected taxa are shown during the first and last sampling events. A reduction in abundance is shown with grey text.

Dominant species present in first sample		Common species present in last sample
	Silver Carp not detected	
Gizzard Shad		Gizzard Shad
White Bass		White Bass
Green Sunfish		Green Sunfish
Largemouth Bass		Largemouth Bass
Smallmouth Buffalo		Smallmouth Buffalo
Bluegill		Bluegill
	Silver Carp in low abundance	
Flathead Catfish		Flathead Catfish
Silver Carp		Silver Carp
Gizzard Shad		Gizzard Shad
Bluegill		Bluegill
Shortnose Gar		Shortnose Gar
Channel Catfish		Channel Catfish
White Crappie		White Crappie
Bigmouth Buffalo		Bigmouth Buffalo
Common Carp		Common Carp
	Silver Carp in moderate abundance	
Silver Carp		Silver Carp
Bowfin		Bowfin (abundance reduced)
Gizzard Shad		Gizzard Shad (abundance reduced)
Smallmouth Buffalo		Smallmouth Buffalo
Bluegill		Bluegill
Bigmouth Buffalo		Bigmouth Buffalo
	Silver Carp in high abundance	
Silver Carp		Silver Carp
Sauger		
Gizzard Shad		
White Bass		
Bluegill		
Green Sunfish		

### Controlled laboratory experiments

**Fig 3:**
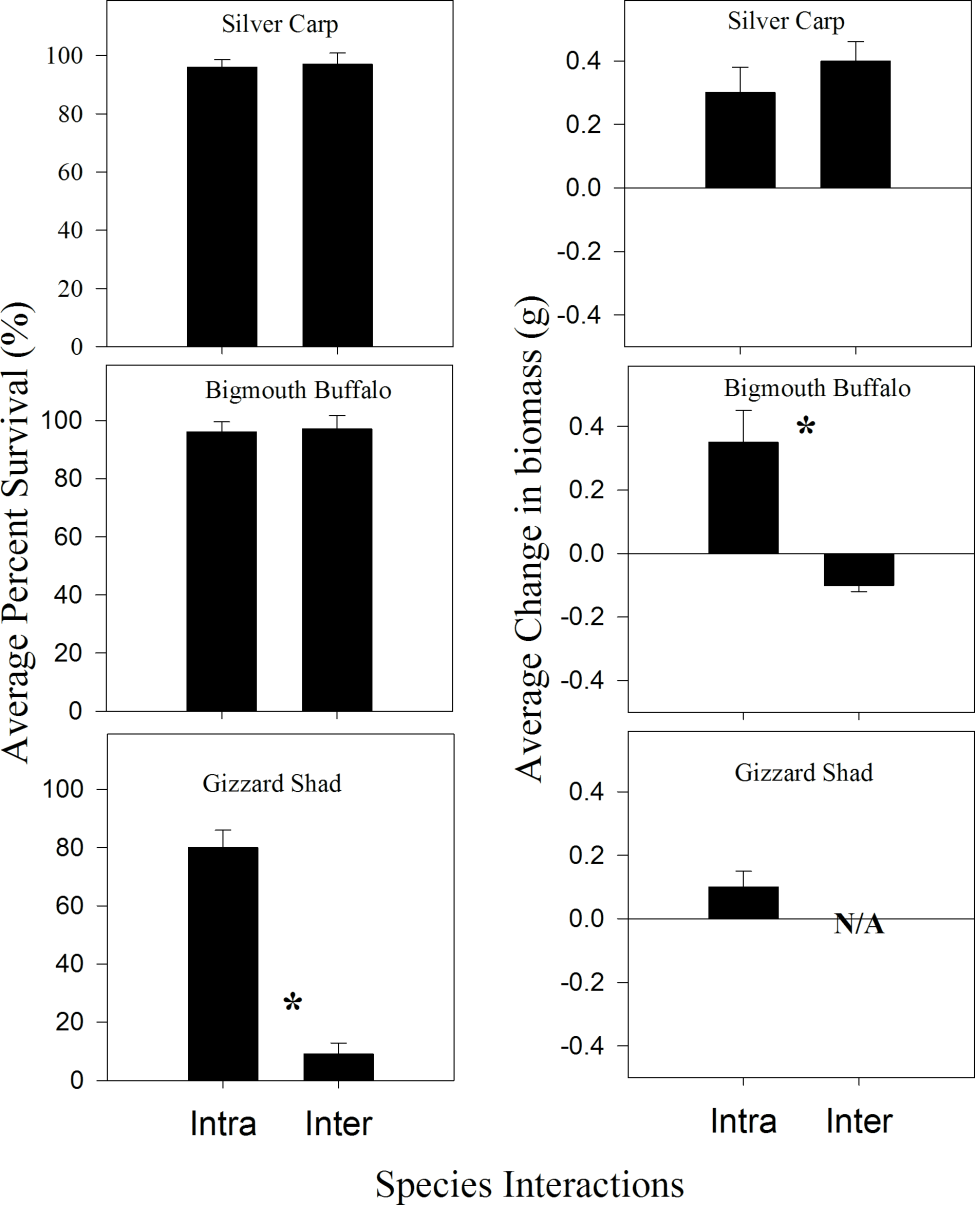
Mean survival and mean growth from both interspecific interaction experiments. Silver Carp *Gizzard Shad [N = 25], and Silver Carp *Bigmouth Buffalo [N = 26]) and intraspecific interaction experiments (Silver Carp*Silver Carp [N = 25], Gizzard Shad*Gizzard Shad [N = 25], and Bigmouth Buffalo*Bigmouth Buffalo [N = 25]). Because Gizzard Shad survival was low, Gizzard Shad growth within the interspecific group was not evaluated (N/A = too few samples to evaluate growth. * Denotes treatments were statistically different, P <0.05. Error bars represent standard error.

Survival did not significantly differ between intraspecific and interspecific interactions for Silver Carp and Bigmouth Buffalo (both comparisons, *P* > 0.05); though, Gizzard Shad survival varied between intraspecific and interspecific treatments (P < 0.05). Gizzard Shad survival was higher in the presence of conspecifics relative to heterospecific interactions (Fig 3). In terms of growth, Silver Carp growth remained consistent between intraspecific and interspecific treatments. However, Bigmouth Buffalo growth within the intraspecific treatment was significantly higher than growth within the interspecific treatment (P < 0.05). Because Gizzard Shad survival was low, Gizzard Shad growth within the interspecific group was not evaluated (too few samples to evaluate growth). Overall, Silver Carp, Bigmouth Buffalo, and Gizzard Shad had high survival when in presence of conspecifics, but in the presence of Silver Carp, Gizzard Shad had low survival and Bigmouth Buffalo exhibited negative growth.

## Discussion

This investigation has undertaken three separate but interconnected approaches (using both applied and basic research) that suggest Silver Carp are deleteriously influencing native fishes. The dramatic increases in CPUE and relative abundance of Silver Carp in the Mississippi River Basin corresponds with prior studies suggesting invasive species thrive in newly inhabited areas [13], [24]. These increases in abundance of Silver Carp were followed by simultaneous declines in Gizzard Shad and Bigmouth Buffalo. We also noted declines in body condition of native fishes after Silver Carp invaded the Mississippi River Basin. Previous investigations [13], [19] and our study results have suggested that the increase of invasive species often coincides with a decline of native fauna. These declines have been attributed to the invasive species creating a limited resource (e.g., prey resources), adversely influencing the native species. This study provides strong cautionary evidence that the high abundance or biomass of the efficient filter- feeding invasive Silver Carp may be creating a limited prey resource for native fishes in the Mississippi River Basin. Potential competition for food resources between Silver Carp and native planktivores has been documented in Germany, China, India, and the Middle East [20], [49–51]; however, this may not always occur [52].

To further evaluate the influence of Silver Carp on native fishes and because of the synergistic relationship between the floodplain and the main river, investigating and evaluating the native and non-native fish species interactions in the floodplain lakes was necessary. We have shown that in newly created floodplain lakes following a major flood event, high Silver Carp abundance can drastically alter native fish communities. Gause [53] determined that species having identical ecological requirements likely compete for the same resources. In situations where ecological niche overlap occurs, the species with greatest competitive tenacity excludes inferior (e.g., less fit) competitors from the sympatric area. Although we did not test for competitive interactions in the floodplain lakes, it is likely Gause’s rule was in-part structuring the relationship we observed. Deleterious interspecific interactions such as competition and eventual competitive exclusion between invasive planktivores and native fishes typically occur when plankton resources become limited [34]. Thus, we suggest that one potential mechanism is competition for food; however, other competitive advantages may exist for Silver Carp [9], [54]. In both the river and the floodplain, our results suggest native fishes are inferior to Silver Carp in the Mississippi River basin. This likely relates back to the exceedingly high abundance or biomass of Silver Carp coupled with their effective filter feeding ability.

Invasive planktivorous species such as Silver Carp are likely influencing native fishes. We have shown quantitatively that Bigmouth Buffalo, Gizzard Shad, and likely other native fishes in the Mississippi River and its floodplain may be at great risk given the abundant, aggressive, invasive filter feeders. Previous field and laboratory studies have shown Paddlefish (*Polyodon spathula)*, Bigmouth Buffalo, and Gizzard Shad in the Mississippi, Missouri, and Illinois rivers may be at risk of competition with Silver Carp [18], [19], [55]. The experimental component of our study further elucidates competition is structuring the relations among invasive carp and native fishes observed in the field components of this study. Specifically, deleterious interactions are likely occurring between Silver Carp and native fishes in the Mississippi River and its floodplain. Despite the damaging effects on fishes occurring at high Silver Carp abundance, our data suggests that low density of Asian Carp is not necessarily a death knell for native fishes. The injurious responses in fish communities are associated with high Silver Carp densities and not mere presence. Thus, Silver Carp could be reduced by developing an integrated pest management program, in turn allowing native fishes persistence. That being said, our study was very simplistic (e.g., qualitative effects at the community level and single species statistical evaluations) in design and statistical approach used. Future efforts should be undertaken to evaluate the effects of Asian carp on the fish community of the Mississippi River and its associated floodplain fish community using a multivariate statistical approach.

In order to develop and implement successful integrated pest management strategies for any nuisance species, a necessary first step is to incorporate all the current scientific information such as basic biology, ecology, interactions with the environment, predictive models, alternative control tactics, and economic impacts [12]. Our study has provided additional information (i.e., influence of Asian Carp on native fishes) that will be needed for feeding the integrated pest management strategy on Asian Carp. In the future, it will be imperative for integrated pest management strategies designed for Asian carp to utilize multiple tiers including prevention actions, early detection measures, monitoring, and containment or control tools to exploit opportunities for Asian carp population management [7].

Based on the data provided in this study the need for an integrated pest management plan is obvious; however, the evaluation of an Integrated Pest Management approach to Silver Carp has yet to be completed. Therefore, the next step in Asian carp control will be to evaluate the feasibility of an integrated pest management program. If an Integrated Pest Management Program were feasible, we suggest using a combination of methods to manage Asian Carp because this multi-tiered approach has been demonstrated to be most effective and provides the most long-term management goal. Using a multi-tiered approach should include control and eradication efforts. Control or eradication efforts may include chemical (e.g., algal attractants, pheromones, piscicides), biological (e.g., bolstering native piscviores), cultural (e.g., habitat manipulation), and mechanical (e.g., commercial harvest); [11], [55–57] removal strategies. Following the mandate by congressional policy to regulate Asian carp in the Mississippi River Basin, this study provides novel scientific information to develop a proactive approach (i.e., integrated pest management) to reduce or prevent further expansion of Asian carp. There are numerous studies being conducted or that could be conducted in the future to further evaluate techniques to slow or stop the expansion of Asian Carp. Techniques that are currently under evaluation are CO2 chambers (Mississippi River Lock Chambers), sound frequencies that only affect Asian Carp, bubble barriers, electric barriers, among others. These techniques could be added to an Integrated Pest Management Program to make the Program more efficient and effective.
